# Impact of Obesity on Ceftriaxone Efficacy †

**DOI:** 10.3390/diseases8030027

**Published:** 2020-07-09

**Authors:** Katie E. Barber, J. Taylor Loper, Austin R. Morrison, Kayla R. Stover, Jamie L. Wagner

**Affiliations:** 1 Department of Pharmacy Practice, University of Mississippi School of Pharmacy, Jackson, MS 39216, USA; kbarber@umc.edu (K.E.B.); tloper11@gmail.com (J.T.L.); amorri14@hfhs.org (A.R.M.); kstover@umc.edu (K.R.S.); 2 Division of Infectious Diseases, University of Mississippi Medical Center, Jackson, MS 39216, USA

**Keywords:** obesity, ceftriaxone, clinical failure, Gram-negative infections

## Abstract

**Background**: Ceftriaxone has standard, set dosing regimens that may not achieve adequate serum concentrations in obese patients compared to non-obese patients. The purpose of this study was to evaluate the effect of obesity on ceftriaxone efficacy when used as definitive monotherapy to treat infections. **Methods**: This retrospective cohort included adult inpatients treated with ceftriaxone monotherapy for ≥72 h between July 01, 2015–July 31, 2017. Patients were excluded if their infection lacked source control within 72 h or if they had polymicrobial infections requiring more than one antibiotic for definitive therapy. The primary outcome was the rate of clinical failure between obese versus non-obese patients, defined as a composite of (1) change in definitive therapy > 72 h due to clinical worsening; (2) residual leukocytosis (white blood cell count (WBC) > 10 × 10^9^/L) > 72 h after treatment initiation; (3) presence of a fever (single temperature > 100.9 °F) > 72 h after treatment initiation; or (4) readmission within 30 days due to re-infection with the same organism. **Results**: A total of 101 patients were included in the study: 39 obese and 62 non-obese. The most common indications for ceftriaxone were urinary tract (52.5%), respiratory tract (24.8%), and bloodstream (24.8%) infections. The most commonly isolated organisms were Escherichia coli (48.5%) and Klebsiella species (15.8%). Most patients received 1g every 24 h. Clinical failure was observed in 61.5% of obese patients versus 40.3% of non-obese patients (*p* = 0.038). **Conclusion**: Obese patients treated with ceftriaxone were more likely to experience clinical failure when compared to non-obese patients. Further analyses are warranted to determine if weight-based dosing is required in obese patients treated with ceftriaxone.

## 1. Background

Over the last 10 years, there has been a significant increase in the prevalence of obesity in adults in the United States [1,2]. It is estimated that 37.9% of adults are obese (BMI ≥ 30.0 kg/m^2^), and 7.7% are morbidly obese (≥40 kg/m^2^) [1,2]. These patients are at an increased risk of infection and poor therapeutic outcomes when compared to average weight patients [3]. In addition, pharmacokinetics of medications are impacted due to excessive adipose tissue and increased glomerular filtration rates, so it can be challenging to achieve optimal therapeutic results based on package insert dosing information [4,5,6]. Because the U.S. Food and Drug Administration (FDA) does not require specific trials to assess therapeutic products in obese patients, there is a paucity of data regarding appropriate dosing in this population. As a result, clinicians are increasingly faced with the challenge of dosing medications in obese patients.

Ceftriaxone has a standard, set dosing of 1–2 g daily being commonly used as monotherapy for a variety of infections. Considering the information above, there is a risk that obese patients are not receiving appropriate ceftriaxone dosing. The purpose of this study was to evaluate the effect of obesity on ceftriaxone efficacy when used as definitive monotherapy to treat infections.

## 2. Methods

### 2.1. Study Design, Setting, Patient Population

The Investigational Review Board-approved study was a retrospective cohort that included adult inpatients who were treated with ceftriaxone monotherapy for ≥72 h from July 01, 2015–July 31, 2017. The exclusion criteria consisted of a lack of source control within 72 h and polymicrobial infections requiring more than 1 antibiotic for definitive therapy. The primary outcome was the rate of clinical failure between obese versus non-obese patients. Secondary outcomes included the following: determination of clinical failure risk factors, 30-day inpatient all-cause mortality, and 30-day hospital readmission.

### 2.2. Study Variables and Definitions

The following data were collected: patient demographics, empiric and definitive antimicrobial therapy used, pertinent laboratory values, and data relating to possible risk factors for treatment failure, microbiology, hospital length of stay, discharge disposition, hospital readmission within 30 days of discharge, and inpatient mortality within 30 days of administration of therapy. Clinical treatment failure was defined as a composite of (1) change in definitive therapy > 72 h due to clinical worsening; (2) residual leukocytosis (white blood cell count (WBC) > 10 × 10^9^/L) > 72 h after treatment initiation; (3) presence of a fever (single temperature > 100.9 °F) > 72 h after treatment initiation; or (4) readmission within 30 d due to re-infection with the same organism. Obesity was defined as BMI ≥ 30.0 kg/m^2^.

### 2.3. Statistical Analysis

The study endpoints were examined using descriptive and inferential statistics. Statistical analysis was performed using SPSS software version 24.0 (IBM, Chicago, IL, USA). Categorical data were analyzed using chi-square or Fisher’s exact test, and continuous data were analyzed using Student’s t-test or the Mann–Whitney U test, as appropriate. An alpha of 0.05 was deemed statistically significant.

## 3. Results

A total of 101 patients were included in the study: 39 obese and 62 non-obese. The median age was 62 (IQR 51–71) years old, and 56% of the population was male. Other than weight ((103 vs. 66 kg (*p* ≤ 0.001); BMI (36 vs. 23 mg/kg^2^ (*p* < 0.001)), there were no differences in baseline comorbidities (Table 1). The majority of patients had previous exposure to empiric antimicrobial therapy prior to ceftriaxone initiation. The most commonly utilized agents were vancomycin and beta-lactamase inhibitor combinations with a median duration of 3 days prior to the initiation of ceftriaxone. The most common indication for antimicrobials were urinary tract (52.5%) followed by respiratory (24.8%) and bloodstream (21.8%) infections. The most commonly isolated organisms were *E. coli* (n = 49; 48.5%) and *Klebsiella* species (n = 16; 15.8%) with no differences between groups. Other organisms isolated included *Streptococcus* species (n = 13; 12.9%), *Proteus* species (n = 11; 10.9%), methicillin sensitive *Staphylococcus aureus* (n = 10; 9.9%), *Citrobacter* species (n = 5; 5%), *Enterobacter* species (n = 2; 2%), other *Staphylococcus* species (n = 2; 2%), and other Gram-negative aerobes (n = 16; 15.8%). All organisms were susceptible to ceftriaxone. A majority of patients received ceftriaxone 1 g (63.4%) every 24 h (94.1%). While there were no statistically significant differences in dosing regimens between groups, obese patients were numerically more likely to receive 2 g of ceftriaxone (46.2% vs. 30.6%; *p* = 0.115). Patients received a median duration of therapy of 5 (IQR 4–7) days (*p* = 0.679).

Clinical failure was observed in 61.5% of obese patients versus 40.3% of non-obese patients (*p* = 0.038) (Table 2). The median hospital length of stay was 14 days, with obese patients experiencing an additional 1.5 days in the hospital compared to non-obese patients (*p* = 0.478). In-patient mortality was more than double in obese patients at 12.8% versus 4.8% (*p* = 0.255). Readmission rates did not differ between patients in either group.

## 4. Discussion

Irrespective of the source of infection or organism isolated, obese patients treated with ceftriaxone were more likely to experience clinical failure when compared to non-obese patients. The primary reasons for clinical failure in the obese group was the addition of a second antibiotic and unresolved leukocytosis. A study by Herishanu et al. noted that obese patients were more likely to have a low-grade reactive leukocytosis [7], which could explain the findings of persistent leukocytosis and possibly the addition of a second antibiotic in our cohort. Clinically, unresolved leukocytosis is commonly a driver for perceived treatment failure. Additionally, these patients were more than twice as likely to die in the hospital and had a prolonged (1.5 d) length of stay.

There are several pharmacokinetic and clinical studies that illustrate that standard dosing of cephalosporins may be inadequate [8,9,10,11,12]. Studies with cefazolin have led to recommendations for increased dosing strategies in patients over 120 kg [8,9], and second-generation cephalosporins have also demonstrated suboptimal target attainment in obese and morbidly obese patients [10]. Similarly, in a case-control study of critically ill obese patients, ceftazidime and cefepime demonstrated lower serum concentrations in these patients [11]. Lastly, although ceftaroline produces lower plasma concentrations in obese versus non-obese patients [12], the probability of target attainment for this agent remains achievable. Interestingly, no data are available assessing the pharmacokinetics of ceftriaxone in an obese patient population.

Our study is not without limitations. First, this study was performed at a single center. However, over 100 patients were included with various sources of infection. Additionally, follow-up data may be incomplete. With multiple hospitals and clinics in the area, and death certificates not assessed, we could only determine a 30-day disposition for those that were readmitted to our facility and clinics.

Higher rates of clinical failures were observed in our obese patient population on ceftriaxone therapy compared to non-obese patients. With data supporting higher dosing required for other classes of cephalosporins in obese patients, larger study populations and further pharmacokinetic analyses are warranted to determine if similar recommendations should be made for ceftriaxone.

## Figures and Tables

**Table 1:** Patient demographics.

Variable	Total	Obese	Non-Obese	*p*-Value
Presented as #(%) or Median (IQR)	(n = 101)	(n = 39)	(n = 62)	
Age	62 (51–70.5)	62 (53–70)	62 (50.8–74.5)	0.761
Sex, male	56 (55.4)	24 (61.5)	32 (51.6)	0.329
Race				
Caucasian	30 (29.7)	11 (28.2)	19 (30.6)	0.794
African American	69 (68.3)	27 (69.2)	42 (67.7)	0.876
Hispanic	2 (2)	1 (2.6)	1 (1.6)	1.000
Serum creatinine, mg/dL	0.99 (0.64–1.97)	1.05 (0.7–1.97)	0.88 (0.61–1.82)	0.319
Weight, kg	80.2 (63.5–98)	103 (95.4–120)	66.25 (58.6–76.9)	<0.001
BMI, mg/kg^2^	27.3 (22.3–32.9)	35.5 (31.2–41)	22.95 (20.8–26)	<0.001
Comorbidities				
Hypertension	75 (74.3)	33 (84.6)	42 (67.7)	0.059
Congestive heart failure	17 (16.8)	10 (25.6)	7 (11.3)	0.061
Cerebrovascular disease	28 (27.7)	10 (25.6)	18 (29)	0.711
Chronic pulmonary disease	23 (22.8)	10 (25.6)	13 (21)	0.586
Connective tissue disease	12 (11.9)	7 (17.9)	5 (8.1)	0.205
Uncomplicated diabetes	24 (23.8)	11 (28.2)	13 (21)	0.405
Moderate-severe CKD	20 (19.8)	9 (23.1)	11 (17.7)	0.512
Charlson score	2 (1–4)	3 (1–5)	2 (1–4)	0.293
Healthcare-associated infection	18 (17.8)	5 (12.8)	13 (21)	0.298
Diagnosis *				
Central nervous system	1 (1)	0 (0)	1 (1.6)	1.000
Bloodstream	22 (21.8)	8 (20.5)	14 (22.6)	0.806
Bone/joint	2 (2)	1 (2.6)	1 (1.6)	1.000
Infective endocarditis	1 (1)	0 (0)	1 (1.6)	1.000
SST/Wound	4 (4)	1 (2.6)	3 (4.8)	1.000
Respiratory	25 (24.8)	11 (28.2)	14 (22.6)	0.524
Intra-abdominal	2 (2)	1 (2.6)	1 (1.6)	1.000
Urinary tract/GYN	53 (52.5)	21 (53.8)	32 (51.6)	0.827

BMI = body mass index; CKD = chronic kidney disease; SST = skin and soft tissue; GYN = gynecological. * More than 1 diagnosis could exist per patient.

**Table 2:** Clinical outcomes.

Variable	Total	Obese	Non-Obese	*p*-Value
Presented as #(%) or Median (IQR)	(n = 101)	(n = 39)	(n = 62)	
Hospital length of stay	14 (8.5–20.5)	15 (8–25)	13.5 (8.8–20.3)	0.478
Discharge disposition				
Died during hospitalization	9 (8.9)	5 (12.8)	4 (6.5)	0.302
Home	51 (50.5)	20 (51.3)	31 (50)	0.900
Skilled nursing facility/rehabilitation	35 (34.7)	11 (28.2)	4 (38.7)	0.280
Hospice	3 (3)	2 (5.1)	1 (1.6)	0.557
Another hospital	3 (3)	1 (2.6)	2 (3.2)	1.000
Treatment Failure	49 (48.5)	24 (61.5)	25 (40.3)	0.038
Readmission in 30 days due to reinfection	11 (10.9)	3 (7.7)	8 (12.9)	0.523
2nd antibiotic added	26 (25.7)	14 (35.9)	12 (19.4)	0.064
Leukocytosis	39 (38.6)	21 (53.8)	18 (29)	0.013
Fever	12 (11.9)	6 (15.4)	6 (9.7)	0.529
30-day inpatient all-cause mortality	8 (7.9)	5 (12.8)	3 (4.8)	0.255
30-day readmission				
No readmission	83 (82.2)	33 (84.6)	50 (80.6)	0.612
Readmitted; infection-related	13 (12.9)	4 (10.3)	9 (14.5)	0.534
Readmitted; non-infection-related	5 (5)	2 (5.1)	3 (4.8)	1.000
